# COVID-19: Are reinfections a global health threat?

**DOI:** 10.3126/nje.v11i1.34903

**Published:** 2021-03-31

**Authors:** Indrajit Banerjee, Jared Robinson, Brijesh Sathian

**Affiliations:** 1, 2 Sir Seewoosagur Ramgoolam Medical College, Belle Rive, Mauritius; 3 Geriatrics and long term care Department, Rumailah Hospital, Hamad Medical Corporation, Doha, Qatar

## Background

The SARS-CoV-2 virus has plagued the planet and caused unfathomable disruptions in every facet of our lives [1]. The undue and unforeseen losses as a consequence of this pandemic will take years to quantify and even longer to curtail. This phenomenon creates an innate pressure on humans as it synergistically charges the environment to propel the movement of restarting enterprise and life as it was before the pandemic in order to curb this “loss”[2].

A plethora of hurdles however are still in question. Firstly, the re-opening of enterprise and a country has to be done in a safe manner. To overcome this hurdle the development of multiple vaccinations to this novel virus has been streamlined [3].

The greatest matter in question however, is the efficacy of these vaccines and whether they will be the answer to allow a safe re-ignition of global enterprise as well as countries as multiple cases of reinfections have been recorded, namely reinfections post recovery from the disease [4,5].

### First reinfection

The first case of reinfection by the SARS-CoV-2 virus was recorded 4.5 months post the initial infection which occurred in March of 2020. The patient being a 33-year-old man from Hong Kong who after undergoing genome sequencing was found to have been infected by two different strains with different genome sequences. It was discovered that a total of 24 nucleotides differences existed between the first and second viral infection. In addition to these differences a truncation of the ORF8 protein was noted in the initial infection which was not present in the latter infection. The above findings suggesting that the acquired immunity gained from the wild type may not be viable and effective against various strains of the virus [6].

### Reinfections on a large scale

A study conducted by Laith J and team, tracked 43044 SARS-CoV-2 positive patients for a median of 16.3 weeks. It was found that the reinfection rate amounted to 0.66/10 000 persons. The natural immunity to these reinfections was calculated to have an efficacy of greater than 90%. In addition to proving that reinfections do occur the study concluded that reinfections were less severe than primary infections and none of the reinfections amounted to death. As the pandemic has spread across the planet more data has become available and thus has allowed for far more accurate projections and has allowed for a far greater understanding of the behaviour of the virus. It is thus evident that the risk of reinfection with COVID-19 is quantified to a much smaller degree by Laith J and team as opposed to Parry Jane [4].

A startling series of events is playing out in Brazil, a study of blood donors indicated that 76% of the population had contracted the virus by the month of October 2020. This figure theoretically crosses the lower limit for the induction of herd immunity which should start yielding results at 67% (of a population being infected). It is thus evident by virtue of the fact that there was an outbreak and rampant increase in cases in early 2021 that the immunity conferred to the Brazilians was not protective in nature and did not prevent reinfections. This is of an international health concern as the P.1 variant is able to re-infect individuals, thus posing a risk to all patients both uninfected and convalescent [7].

### Natural immunity to COVID-19

Sheila F. Lumley and team conducted testing of anti-spike IgG on 12541 healthcare workers and subsequently quantified the relative levels of IgG to that of reinfections. 11364 of the workers tested negative whereas 1265 were positive, 88 of whom in which seroconversion had occurred. A base level of anti-spike IgG was determined in each of the individuals. It was found that the presence of anti-spike/anti-nucleocapsid IgG antibodies correlated with a pronounced reduced risk of a reinfection with SARS-CoV-2 within a period of 6 months. It was additionally evident that healthcare workers with a higher titre of the antibodies displayed milder symptoms or were asymptomatic. It is thus evident that natural antibodies produced against the virus do help combat both reinfection as well as mediate the symptoms of the virus. This finding is poignant and of the upmost value as it acts as a positive yardstick and indicator for the development and efficacy of vaccines against SARS-CoV-2. It must be noted however, that the true duration and extent to which natural acquired immunity offers protection is not well understood and only time and further study will provide the necessary data to draw a sound scientifically based conclusion [8].

It must be noted that this phenomenon of reinfection does not occur exclusively in the SARS-CoV-2 group, but reinfections are a well-established trait in various subsets of Coronaviridae. The Coronaviruses 229E, HKU1, OC43, as well as NL63 cause seasonal reinfections due to the evanescent nature of the established host immunity. It is this evanescence and short lived nature of the host immunity that may be implicated in the reinfections occurring with the SARS-CoV-2 virus [9].

### Theories behind reinfection

A study by Mohammad Asim and team on using the metabolomic approach to both combat and better understand the virus has based the likelihood of reinfection on an individual basis, namely if an individual has some degree of metabolomic dysfunction it is evident that the particular individual in question will have a greater likelihood of reinfection. Based on this model the quantification of the likelihood of reinfections can be better understood and thus curtailed via the correction of micro imbalances within an individual’s physiology [10].

### Reinfections and viral mutations

As in the first recorded case of reinfection, the viral genomes and subsequently the viral proteins of the two infections were markedly different. Mutations among viruses are common and often are detrimental to humans and make the treatment thereof complex and difficult, as in the case of (HIV) human immunodeficiency virus where rapid mutations occur frequently. The influenza virus periodically undergoes antigenic drift and shift, in antigenic drift minor mutations and alterations occurs which allow the natural immunity to be effective against the new variant of the virus, however when antigenic shift occurs it causes the natural acquired immunity to no longer be viable and effective and thus leads to an epidemic. A similar picture could be occurring with COVID where antigenic shift is occurring more rapidly and thus the already acquired natural immunity is no longer effective against the new variant, thus creating the breeding ground for re-infections. It is however vital for extensive sampling in reinfection cases so as for the comparison of the viral genome between the initial and the secondary infection. This data being used to determine whether the reinfection was caused via failure of the acquired natural immunity against the virus. It will also establish whether the reinfection is due to an entire new mutant, where existing immunity is noneffective and will not suffice in mounting an adequate immune response [11,12,13,14].

### Reinfections, mutations and their bearing on immunization

Immunization has been the gold standard method to both eradicate and diminish the impact caused by the SARS-CoV-2 pandemic. The question and concern however is vaccine efficacy and whether the vaccines that have been synthesized and that are available on the market will be effective against the new mutations. The various mutations of the virus will dictate reinfection rates as well as immunization efficacy. The B.1.351 variant of SARS-CoV-2 AKA “the South African strain” has caused great concern for the international community as a whole and poses a threat to other nations already undergoing mass scale immunization as it has been found that the B.1.351 variant is more resistant. Vaccines such as Oxford AstraZeneca have been labelled as to have “limited efficacy” against this strain and thus has caused the South Africans to opt for alternate vaccines such as Pfizer vaccine as well as Johnson and Johnson. It must be noted however that the Oxford AstraZeneca is not totally ineffective against the strain, as in a small trial of 2026 people in South Africa it was noted that no patient died nor was hospitalized. The pharmaceutical company is well underway in the process of adapting the vaccine to be effective against the new strain [15,16, 17].

### Infections post immunization

After the initiation of the immunization with both the Moderna mRNA-1273 vaccine and the Pfizer BNT162b2 vaccine, cases of infection with COVID-19 have been reported. Vaccinated healthcare workers in America underwent periodic testing via PCR. A large cohort of data was collected between December 2020 and the beginning of February 2021. A total of 36 659 individuals received the initial dose and 28 184 completed the immunization. 379 individuals tested positive for COVID within the first fortnight after the initial dose. 37 positive cases were recorded after the second dose; 22 of which were registered within the first week after completing the immunization. The decrease of infections after the second dose of the vaccine is promising, however it is clearly evident that infections in an immunized individual is not only a possibility but is occurring and thus the adherence to protocols and minimal exposure should be exercised around the immunization period until a full antibody response has been mounted. It is also evident that new vaccines will have to be synthesized in order to target the numerous variants [18].

### Dataset challenges in calculating reinfections

The likelihood of underreporting of actual infections and subsequent reinfections critically disrupts the dataset and hinders the generation of accurate projections. Due to the novel nature of this virus all reliable scientific evidence has to undergo peer review, this specific and verifiable process requires reputable evidence such as PCR test results as well as genome sequencing etc. The innate challenges that this yields is that many individuals who were infected in the initial wave of the virus were never tested via PCR. This naturally creates a dearth within the dataset and therefore it can be suspected that the current figures are only a fractional representation of the true global dataset [9].

## Conclusion

It is evident that reinfections of the SARS-CoV-2 virus can be attributed to two major benefactors. The first being a decrease in production or failure of natural acquired immunity thus leading to the reinfection by the same strain of the virus rendering the person ill and or asymptomatic. The second benefactor is that of mutations. Mutations of the virus will predispose to reinfections as the already established naturally acquired immunity is not beneficial or useful to combat a mutated strain. It is thus poignant for cases of reinfection to be investigated thoroughly as to determine whether the reinfection was caused due to an innate lack of immunity or due to a new strain where no prior immunity existed. It must however be noted that the cases of reinfections are a rather rare event and even if a reinfection does occur, the case if usually mild and or asymptomatic. The impact of reinfection on immunization at this point of time is difficult to quantify however, initial reports do show positive signs even in cases where the vaccine is not fully effective against a mutated strain.

## References

[1] Banerjee I Robinson J Kashyap A Mohabeer P Shukla A Leclézio A. The changing pattern of COVID-19 in Nepal: A Global concern- A Narrative Review. Nepal J Epidemiol. 2020 6 30;10(2):845-855. https://doi:10.3126/nje.v10i2.29769 PMid: ; PMCid: . 32874698 10.3126/nje.v10i2.29769 PMC7423402

[2] Banerjee I Robinson J Kashyap A Mohabeer P Sathian B. COVID-19 and Artificial Intelligence: the pandemic pacifier. Nepal J Epidemiol. 2020; 10(4); 919-922. 10.3126/nje.v10i4.33334 PMid: PMCid: 33495709 PMC7812328

[3] Sharma O Sultan AA Ding H Triggle CR. A Review of the Progress and Challenges of Developing a Vaccine for COVID-19. Front Immunol. 2020 10 14;11:585354. 10.3389/fimmu.2020.585354 PMid: PMCid: 33163000 PMC7591699

[4] Abu-Raddad LJ Chemaitelly H Coyle P. SARS-CoV-2 reinfection in a cohort of 43,000 antibody-positive individuals followed for up to 35 weeks. medRxiv. 2021 1 1. 10.1101/2021.01.15.21249731 PMCid:

[5] Nainu F Abidin RS Bahar MA. SARS-CoV-2 reinfection and implications for vaccine development. Hum Vaccin Immunother. 2020 12 1;16(12):3061-3073. https://doi:10.1080/21645515.2020.1830683 PMid: . 33393854 10.1080/21645515.2020.1830683 PMC8641611

[6] Parry J. Covid-19: Hong Kong scientists report first confirmed case of reinfection. BMJ. 2020 8 26;370:m3340. https://doi:10.1136/bmj.m3340 PMid: . 32847834 10.1136/bmj.m3340

[7] Sabino EC Buss LF Carvalho MPS. Resurgence of COVID-19 in Manaus, Brazil, despite high seroprevalence. Lancet. 2021 2 6;397(10273):452-455. https://doi:10.1016/S0140-6736(21)00183-5 PMid: ; PMCid: . 33515491 10.1016/S0140-6736(21)00183-5 PMC7906746

[8] Lumley SF O’Donnell D Stoesser NE. Antibody status and incidence of SARS-CoV-2 infection in health care workers. New England Journal of Medicine. 2020 12 23. 10.1056/NEJMoa2034545 PMid: PMCid: 33369366 PMC7781098

[9] Boyton RJ Altman NDM. Risks of SARS-CoV-2 reinfection after natural infection. The Lancet. Online first. 2021. 10.1016/S0140-6736(21)00662-0 PMC7969128 33743219

[10] Asim M Sathian B Banerjee I Robinson J. A contemporary insight of metabolomics approach for COVID-19: Potential for novel therapeutic and diagnostic targets. Nepal J Epidemiol. 2020 12 31;10(4):923-927. 10.3126/nje.v10i4.33964 PMid: PMCid: 33495710 PMC7812325

[11] Iyengar KP Jain VK Ish P. COVID-19 reinfection - An enigmatic public health threat. Monaldi Arch Chest Dis. 2020 11 30;90(4). 10.4081/monaldi.2020.1596 PMid: 33305559

[12] Nachmias V Fusman R Mann S Koren G. The first case of documented Covid-19 reinfection in Israel. IDCases. 2020;22:e00970. 10.1016/j.idcr.2020.e00970 PMid: PMCid: 33029476 PMC7528892

[13] Biswas A Bhattacharjee U Chakrabarti AK Tewari DN Banu H Dutta S. Emergence of Novel Coronavirus and COVID-19: whether to stay or die out? Crit Rev Microbiol. 2020 3;46(2):182-193. 10.1080/1040841X.2020.1739001 PMid: PMCid: 32282268 PMC7157960

[14] Mustapha JO Abdullahi IN Ajagbe OOR. Understanding the implications of SARS-CoV-2 re-infections on immune response milieu, laboratory tests and control measures against COVID-19. Heliyon. 2021 1 9;7(1):e05951. 10.1016/j.heliyon.2021.e05951 PMid: PMCid: 33490695 PMC7810769

[15] Oxford coronavirus vaccine IS less effective against South African mutant strain found in UK, claim scientists: Small study of just 2,000 patients found some patients got mild or moderate symptoms - but NONE died or were hospitalised. Mail online. [online 2021] [cited 2021 March 21] Available from: URL: https://www.dailymail.co.uk/news/article-9232411/Oxford-Covid-vaccine-effective-against-South-African-mutant-strain-claim-scientists.html

[16] Covid: South Africa halts AstraZeneca vaccine rollout over new variant. BBC News [online 2021] [cited 2021 March 21] Available from: URL: https://www.bbc.com/news/world-africa-55975052

[17] Baraniuk C. Will Delaying Vaccine Doses Cause a Coronavirus Escape Mutant? The Scientist. [online 2021] [cited 2021 March 21] Available from: URL: https://www.the-scientist.com/news-opinion/will-delaying-vaccine-doses-cause-a-coronavirus-escape-mutant--68424?utm_campaign=TS_DAILY_NEWSLETTER_2021&utm_medium=email&_hsmi=109672011&_hsenc=p2ANqtz--TPwtjHgEPz5fDYOluxy0Jh7DXzb04rxOBLoY6TtNo-SOvixq6BVcW59dhWM5fMpkVTCKyL5AqodOqL3Q9_8WsXlMQaw&utm_content=109672011&utm_source=hs_email

[18] Keehner J Horton LE Pfeffer MA. SARS-CoV-2 Infection after Vaccination in Health Care Workers in California. N Engl J Med. 2021 3 23. https://doi:10.1056/NEJMc2101927 PMid: . 33755376 10.1056/NEJMc2101927 PMC8008750

